# Hospital and Institutional News

**Published:** 1919-02-15

**Authors:** 


### HOSPITAL AND INSTITUTIONAL NEWS.

### TEMPORARY VERSUS PERMANENT HOSPITALS.

The war has brought about many changes, one of the most recent being a result of the remarkable success of the treatment of the severest cases in tents, huts, and temporary buildings of various kinds. The complete success of treatment under poor conditions in these types of buildings makes it clearly the duty of every one connected with efforts to add to the existing hospital accommodation in this country, either through small hospitals as memorials, or extensions, or larger hospitals up to 750 beds, which experience shows to be about the maximum number which can be efficiently handled. All hospital managers should give careful thought to this aspect of the question. In the *Encyclopædia Britannica*, latest edition, Volume XIII., is an article under "Hospitals" by Sir Henry Burdett, which contains a forecast of the form in which every great city and populous should organise its hospital system and secure the maximum of efficiency and the best results, at the smallest possible cost. We mention this because the experiences of the war have given a special importance to this forecast, which all, who are interested in new and up-to-date schemes for hospital provision and buildings, including the new Ministry of Health, must find important and well calculated to provide food for thought.

### CARDIFF AND TEMPORARY HOSPITALS.

Colonel Joseph Griffiths, M.A., M.D., M.C., F.R.C.S., commanding First Eastern General Hospital, Cambridge, in the *Western Mail* of the 4th instant, first points out what he describes as the present unsuitability of the local infirmary for housing sick persons. He then proceeds to advocate the erection of temporary hospital buildings and the practical scrapping of the present King Edward VII.'s Hospital, Cardiff, or to put its accommodation to other uses. Now King Edward VII.'s Hospital has been for years, as it has been steadily developed, through the devotion of Colonel Bruce Vaughan, his colleagues and friends, and the men and women of Cardiff, one of the most interesting and instructive exhibitions of voluntary hospital development. The courage with which great problems have been solved, the wonderful financial ability exhibited, and the splendid generosity of, and steady growth in the contributions of all classes of people interested in and using the hospital, when occasion has arisen for the succour of their families and themselves, stand out as a record of encouragement to hospital authorities everywhere for all time. Has Colonel Griffiths read the description of "The Hospital City of the Future" in the *Encyclopædia Britannica*, Volume XIII., because, if he has, his article puts him out of court as a reliable guide in the erection of a new hospital in Cathays Park at a cost not exceeding "£60 to £70 per bed for a building that will adequately serve all the purposes of a hospital." If he has not, we suggest that he should lose no time in referring to the *Encyclopædia Britannica* and mastering the article in question. He will then discover that to provide adequately in the year after the war for the hospital needs of the inhabitants of a city with a population, in round numbers, which approaches 200,000, when every attempt must be made to bring medical treatment and hospital care up to the highest standard, a Hospital City should be the aim and ambition of every community which contemplates scrapping its existing hospital buildings. Cardiff could then create in their place the most perfect, up-to-date, and hygienically complete accommodation, which it is at present possible to provide. Certainly Colonel Griffiths' suggested scheme "to meet the needs of Cardiff and its neighbourhood to-day, and in the immediate future," falls far short of the mark, and is wholly unworthy of the city of Cardiff, which has provided itself already with King Edward VII.'s Hospital, with all its educational and moral advantages that the citizens have derived from its teaching and work. We should be indebted to Colonel Griffiths if he would kindly give us the names of any hospitals which fulfil his conditions, and can be erected at a cost, "which need not exceed at to-day's enhanced prices £60 to £70 per bed for building."

### GUY'S DOUBLE LOSS.

Within the last few days Guy's Hospital has bid farewell to Sir Arbuthnot Lane, senior surgeon, and to Sir W. Hale White, senior physician, both of whom are now promoted to the Consulting Staff. During the war the rule of retiring from the active Staff at the age of sixty was placed in abeyance, and in the case of both Sir Arbuthnot Lane and Sir W. Hale White the offer of extended services was gladly accepted by the hospital authorities. Many old Lane H.S.s accompanied Sir Arbuthnot in his last round, and Mr. Thomas (the last Lane H.S.) and Mr. Mollison expressed the regret felt by the hospital at the loss of one so distinguished, to which was joined a sense of pride that one so world-famous could be claimed as a Guy's man. In expressing his thanks, Sir Arbuthnot said that during his forty-six years at Guy's he had been privileged to be associated with most able men, and fortunate in having as house surgeons "some of the best men England can produce." "The future of surgery," he added, "lies in their hands, and I am content to leave it there."

### ADMIRATION OF SIR W. HALE WHITE.

Animated by a feeling of admiration for their departing chief, there was a large assembly in Addison Ward, Guy's, when Sir W. Hale White's last round took place on February 3. At the conclusion of the round the sense of loss felt by all was voiced by Mr. W. Halstead and Captain C. P. Symonds. Sir Hale White, briefly acknowledging the speeches, referred to his forty-four years' service at Guy's. In placing on record these farewell proceedings, *Guy's Hospital Gazette* says: "These two familiar faces will be sorely missed in the hospital, but they have left behind two memorable influences to add to those of the great ones that pass on from Guy's. We wish to both many more happy and useful years in which reputations may be added to, and venture to hope that as many as possible of the hospital functions will be graced with their presence."

### LEICESTER ROYAL INFIRMARY RETIREMENT.

On the anniversary of his sixtieth birthday, an age at which Surgeons to the Leicester Royal Infirmary retire, opportunity was taken by Mr. T. Fielding Johnson, Junior (High Sheriff), the Chairman of the Board, to entertain Major H. J. Blakesley, F.R.C.S., the Senior Honorary Surgeon, to a complimentary dinner. All his colleagues on the Consulting and Honorary Medical and Surgical Staff were present to bid farewell to their colleague, as also were many personal friends. Major Blakesley's association with the Infirmary covers a period of twenty-nine years, the first eleven of which were spent as assistant-surgeon in charge of out-patients. The great progress in the Institution and in its equipment and service, in which the retiring surgeon's advice and counsel had always been most helpful, were prominent points in the speeches of the evening, and the testimony of his medical and surgical colleagues to the interest which Major Blakesley invariably displayed in the Institution and in the patients was both appropriate and deserved. Doubtless in due course Major Blakesley's name will be added to the list of Consulting Surgeons to the Institution, and he will still retain charge of the department recently established for the treatment of venereal diseases (male clinic), and of course continue his private practice.

### TUBERCULOSIS HOSTEL FOR LONDON.

For some time local authorities in London have been agitating for a hospital where cases of advanced tuberculosis might be sent with the object of removing "dangerous" cases from the community. Such an institution is now proposed, the idea being that it shall be organised by the Greenwich Borough Council and supported by other authorities. The medical officers of the Local Government Board, London County Council, Insurance Committtee and the War Pensions Committee have decided to recommend the authorities they advise to subsidise the new institution to the fullest extent of the capacity contemplated at the rate of £2 per bed per week. The scheme provides for thirty beds. The income is estimated at £3,120 yearly, being twenty L.C.C. patients and ten Insurance Committee patients, at £2 a week each. As the other authorities may be considering similar schemes it may be of interest to add the subjoined statement of expenses: Chief executive tuberculosis officer, £100; Tuberculosis officer as medical officer, £100; Matron, £100; Two charge nurses, £120; Two nurses, £80; Four probationers, £104; One cook, £40; Two servants, two wardmaids, £135; One porter, £80; Rents, rates and taxes, £150; Gas coal and water, £100; Laundry, £50; Food for staff at 12s 6d., £455; Food for patients at 10s., £780; Dispenser, £26; Drugs, £300; Equipment, renewals, £100; Repairs, £100; Interest on sinking fund and capital, £200.

### BOURNEMOUTH'S THANKOFFERING.

Over £800 was raised at Bournemouth on January 25 for the Royal Victoria and West Hants Hospital, as a New Year Thankoffering. The effort was organised by the Bournemouth Hospital Saturday and Sunday Fund, in conjunction with Bournemouth Flag Day Committee. This fine sum, raised in the face of unfavourable weather, bears testimony to the enthusiastic work of the two committees, and is a tribute to the generosity of the people of Bournemouth, who always respond splendidly to an appeal for their own hospital.

### JUBILEE RECORDS.

The Birkenhead and Wirral Children's Hospital, which attains its jubilee this year, should not remain without a written history. A jubilee is Time's reminder that the hour for recording experiences and developments has come, and these have their value in preserving the hospital tradition, without which no voluntary hospital is able to do its best work. Mr. F. W. Archer, the hon. secretary, who has the facts—or most of them—at his fingers' ends, should consider the opportunity provided by the jubilee. There is no reason why a small hospital should not have an interesting history. Jane Austen went no further than the drawing-room for her materials, and the changes in institutional life since 1869, whether on a small or large scale, could not fail to be interesting to any one who dug a little deeper than the statistics of the reports.

### THE HALESOWEN COTTAGE HOSPITAL.

Mrs. H. C. Dickson has presented Mount House, at the foot of Mucklow's Hill, to the committee appointed in the Halesowen district to provide a cottage hospital. Over half the sum in view—namely, £3,000—has now been promised, and an appeal is to be made to equip and maintain the hospital.

### NORTHAMPTON CONVALESCENT HOME SCHEME.

The support shown for Northampton General Hospital is so healthy that a plan is on foot to provide it with a convalescent home. In support of the plan, Dr. Robson has pointed out that there was no such institution in the county at present, and the advantages to patients and those on the waiting-list would be equally great. The workers' contributions have been steady and extensive, and the scheme promises to gain general support.

### SYSTEM OF INVALID RATIONS.

In reply to a letter from Dr. Robert Bell in the *Daily Express* of February 4 on the treatment of applications for invalid rations, the Ministry of Food has published a very clear statement. Applications are dealt with in the first place by local food committees, and those requiring further investigation are submitted to the medical adviser of the Ministry. If these cases be stated as urgent, the local authorities are empowered to grant extra rations for a period of two weeks upon a medical certificate to that effect. These arrangements were made in consultation with the Reference Committee of the Royal College of Physicians and the Royal College of Surgeons, and regulations have been from time to time revised by the same procedure. The working of such a system may in isolated instances create some apparent delay, but provided the matter rests in local hands for the first fourteen days, there should be ample opportunity to obtain meanwhile the opinion of the higher medical consultant. In outline the method commends itself, and we suggest that any failures must have their origin in personal delay rather than inefficiency of the system.

### BRITISH PREVENTIVE MEDICINE.

Professor J. G. Adami, of the Canadian Expeditionary Force, in a lecture before the members of the Royal Institution on February 7, expressed great admiration for the recent work of the R.A.M.C. and the medical men of Great Britain, and explained that, contrary to the prevalent idea, the most notable advance had been made, not in surgery, but in preventive medicine. Through all times continued disease has been the deadliest enemy of the field army, and its removal must be appreciated as fully as any strategical victory. A British Commission disinfected Serbia and practically stopped an extremely serious outbreak of typhus. In the present Canadian Overseas Force, in size approximately equal to the whole British Army of the Boer War, there had been, Professor Adami stated, but 412 hospital typhoid cases and 14 deaths, against 57,684 cases and 8,248 deaths from the same disease during the South African campaign: the use of anti-tetanus serum had reduced the mortality rate to about one per hundred, while in the instance of so-called spotted fever a current report shows that a modern serum reduces the deaths to 12.4 per cent. of infected persons. The lecture abounded with similar instances of success, and leaves one with sanguine hopes for the future.

### LECTURES ON MALARIA.

A series of four excellent lectures is being delivered at King's College Hospital, London, by Colonel Sir Ronald Ross, the Consultant in Malaria at the War Office. The first two have been devoted to original research leading to the discovery that malaria was a living contagion in the blood, and the connection between the mosquito and spread of the disease. Prevention on a large scale is next discussed together with earlier methods of treatment, but in the last two lectures it is understood that the most recent developments in this direction will be given in detail. Coming from so great an authority after a period so replete with world-wide investigations, they should be of the greatest value and interest.

### INFLUENZA'S FAVOURITE VICTIMS.

That epidemics, especially the influenza epidemic, are greater destroyers of life than war receives confirmation from South Africa. Sir Thomas Watt, Acting Prime Minister, stated in the House of Assembly that during the war the total losses on service were 7,089 Europeans, and 1,105 "coloured" and natives. But in two months alone the influenza epidemic carried off 11,736 Europeans, and 127,700 "coloured" and natives. So far as Europeans were concerned, the scourge attacked not the old and the weak, but rather the middle aged and the strong, which is a peculiarity of the epidemic noticeable in other countries it has visited. South Africa, by the way, is on the point of adopting a Public Health measure. That such a step is as much needed in the Union as in this country is made evident by the statement of Sir Thomas Watt, that out of 30,520 would-be recruits medically examined since December 1917, no fewer than 15,000 were rejected, mainly for defective eyesight, poor physique, and heart disease.

### DEATH-DEALING MILK.

Major W. Astor, M.P., the new Parliamentary Secretary of the Local Government Board, who has on many occasions shown that he is impressed with the importance of an abundant supply of hygienic cheap milk in the fight against tuberculosis and the promotion of child welfare, stated at a meeting of the Royal Institute of Public Health that it is "comparatively easy to provide cheap and safe clean milk; the question to a large extent was one of method." He went on to suggest that the price to the producer should be based upon a quality of milk children could safely consume, and that "farmers or retailers who did not take the trouble to provide milk of a standard cleanliness should be paid less than the standard price." It is probably true, as he hinted, that if it were known that "contaminated, dirty, tuberculous or stale milk could only be sold below the usual price," an immediate improvement would follow. But the public would feel more satisfaction if a leader in this crusade against death-dealing milk had suggested punitive measures against the producers and purveyors of the stuff described with so many destructive adjectives. Mere reduction in the scale of profit is a weak weapon. "Dirty, tuberculous or stale milk" foisted upon the public should at least lead to heavy monetary penalties inflicted by a representative of the law.

### THE ORGANISATION OF OVERLAPPING.

In a recent address to the Liverpool Central Relief and Charity Organisation Society the Lord Mayor made two points which deserve general attention. The first was that only 1 per cent. of the inhabitants subscribed to any of the charities in the city. The second point concerned the visiting which is part of the Society's work in compiling the 50,000 family records which it has upon its books. The Society, said the Lord Mayor, was trying to eliminate the multiform visitation of the poor, and the Society did not wish its visitors to be regarded otherwise than as friends, who were not to be asked to perform duties which belonged to the paid officers of a public authority. This statement is encouraging, for the centralisation at which all charity organisation aims is apt to degenerate into red tape and inquisitional visits.

### PATIENTS AS LIFE GOVERNORS

A SUCCESSFUL year's work in connection with the Ross Cottage Hospital is shown by the remarkable fact that seven of those who were admitted as in-patients became life governors at their departure. In addition to this, a working man instructed his executors to sell his cottage and hand the proceeds to the hospital. This appreciation speaks for itself.

### FEWER REGISTERED PHARMACISTS.

FROM the report of the Registrar of the Pharmaceutical Society of Great Britain just issued it appears that during last year there was a decrease of 130 names on the register. As with allied professions, this is attributed to the absence on active service of many students and those males who were in the apprenticeship stage. The loss has, to some extent, been counter-balanced by the increasing number of women taking up the profession of Pharmacy. With the return to more normal conditions the number of entrants for examinations is likely soon to reach the pre-war standard, especially as the Government and the Pharmaceutical Society give assistance to demobilised and wounded members of the Forces who desire to take up or resume the profession of Pharmacy. It is interesting to note that the number of members of the Society (membership being voluntary and apart from registration) is yearly increasing; the total is now about 9,000. The number of registered pharmacists is about 16,000, but some deduction must be made for those not actively engaged in the practice of pharmacy, but who have gone in for medical and analytical degrees.

### THE DEVELOPMENT OF ENGLISH SPAS.

FROM time to time attempts are made to increase the popularity of English spas. The willing analyst provides the necessary testimony to the efficacy of the water; medical men are encouraged to acquaint themselves with its virtues; even the lighter side of the attractiveness of the spa is not forgotten. Cheltenham has now decided to furnish the Montpellier Baths with a complete installation of medical baths, and the Town Council was doubtless influenced by the fact that other spas are considering similar schemes in the hope of retaining some of the visitors who used to flock to the Continent. We suspect that the difficulty in the way of the success of the English spa is largely due to the uncertainty of the English weather, and in part to the fact that no English resort can offer to an Englishman the change of a Continental visit.

### THE INSPECTOR'S SILK HAT.

THE " air of authority " given by a silk hat was the explanation offered by the Liverpool Health Committee to a criticism of its proposal to buy 104 silk hats for its 104 sanitary inspectors. The request came from them, and, said the chairman, amid laughter, they were sometimes mistaken for clergymen. In the case of the women sanitary inspectors, for whom blouses and silk scarves were proposed, the idea was to provide distinctive clothes but not a uniform. The inspectors in the end had to be content with a cap, but, if this is judiciously designed, it might lead them to be mistaken for a police sergeant, and give them an even more exalted air. For there is this force in their plea: that the public has come to distrust the arrival of a nondescript giving attention to gas or meter, light or drains. Municipal officials who come on public business should wear an official cap or some cut of coat to identify them.

### SOME PROMPT HOSPITAL STATISTICS.

THE New Year was not many hours old when the general manager of the General Infirmary, Leeds, Mr. Fred J. Bray, issued his comparative statement of admissions and attendances for the year 1917-18. This return contains much interesting information as to the work of the great Yorkshire hospital, and the figures are arranged in a clear and understandable manner. We notice that in 1918 there were 4,781 operations on in-patients, and 2,250 on out-patients; 3,007 patients were skiagraphed, and 5,611 specimens were examined in the pathological department.

### AN EXTRAORDINARY PROPOSAL

THE lack of co-ordination between Government departments could not be better illustrated than by a suggestion made in a letter of the Pensions Ministry to the Fife Local War Pensions Committee, and discussed at the last meeting of that body. The proposal was regarding the treatment of tuberculous cases, who, it was suggested, should be boarded out in private houses, there to have home comforts and do light jobs about the house and garden. Now surely if the Pensions Ministry had taken competent professional advice, or sent across to the Local Government Board, it could have got the information that consumptives need separating from the homes of the general community rather than sending into them. The suggestion was, of course, negatived, indeed ridiculed.

### THIS WEEK'S DRUG MARKET.

GENERALLY speaking the downward tendency of prices continues and business remains extremely quiet. There are exceptions, however. For instance, a steady business is passing in iodides, mercurials and bismuth preparations, and at present there is no sign of an impending drop in the prices of these. The price of English refined camphor is firmly maintained, and Japanese refined is rather dearer. On the other hand, many of the synthetic drugs are lower, as for example aspirin, salicylic acid, sodium salicylate, benzoic acid, sodium benzoate, barbitone, artificial oil of wintergreen, salol, antifebrin and hexamine. Japanese peppermint oil and menthol are quiet and tend downwards. Bromides are in adequate supply and easier. Eucalyptus oil is fairly plentiful, while the demand has fallen off. Chloral hydrate is cheaper. There is no difficulty in obtaining glycerine, and castor oil of good quality is readily obtainable.

